# A Rare Case of Exaggerated Placental Site Reaction

**DOI:** 10.7759/cureus.90139

**Published:** 2025-08-15

**Authors:** Rajitha D Wijesinghe

**Affiliations:** 1 Gynecological Oncology, National Hospital Kandy, Kandy, LKA

**Keywords:** exaggerated placental site, gestational trophoblastic disease, hcg, invasion, placental site trophoblastic tumor

## Abstract

Exaggerated placental site (EPS) reaction is a rare, poorly understood benign condition associated with persistent and excessive invasion of the uterus by intermediate trophoblasts. It often can mimic placental site trophoblastic tumor and epithelioid trophoblastic tumor. We present a 34-year-old woman with persistent elevation of human chorionic gonadotropin (hCG), who was unsuccessfully treated with chemotherapy for suspected gestational trophoblastic neoplasm. An MRI scan revealed infiltrative disease involving the uterine cervix and lower segment, following which she underwent hysterectomy. Histology was in favor of an EPS reaction, and hCG returned to a nondetectable level after surgery. This case highlights the importance of considering the possibility of EPS reaction in patients presenting with a uterine mass and persistently low levels of hCG despite chemotherapy. However, preoperative confirmation of EPS reaction could be challenging.

## Introduction

Exaggerated placental site (EPS) reaction is a rare but non-neoplastic gestational trophoblastic disease that can often mimic placental site trophoblastic tumor (PSTT) and epithelioid trophoblastic tumor (ETT). This condition is associated with exuberant infiltration of the endometrium and myometrium by intermediate trophoblasts at the site of implantation and is often considered an exaggeration of the normal physiological process. It is worth noting that this is neither an inflammatory nor a neoplastic condition, and diagnosis is essentially histological. Unlike gestational trophoblastic neoplasms, EPS does not demonstrate malignant features such as local organ infiltration or metastasis. This reactive condition can occur following miscarriage or ectopic, molar, or healthy pregnancies [1,2]. Only a handful of EPS cases have been reported, and it is considered that around 1.6% of first-trimester miscarriages result in this benign, often self-limiting condition [3]. While most women are asymptomatic, some present with abnormal uterine bleeding and persistent detectable human chorionic gonadotropin (hCG) levels. This article presents a challenging case of histologically diagnosed EPS, which presented clinically as a gestational trophoblastic neoplasm.

## Case presentation

The presented case is a 34-year-old gravida 3, para 1 with persistently elevated hCG following a first-trimester miscarriage. She was otherwise healthy and did not have any medical comorbidities. Her first pregnancy was a live birth at term by lower segment cesarean section, followed by a surgically managed first-trimester miscarriage two years before the index presentation. No histological assessments were performed.

Her third pregnancy ended as a first-trimester miscarriage, after which she underwent suction dilation and curettage. However, histological examination of the tissues had not been carried out. A self-administered urine pregnancy test was positive at three weeks after the surgical evacuation. Weekly serial hCG monitoring revealed slowly rising levels, after which she was treated with methotrexate (Table 1).

**Table 1:** Human chorionic gonadotropin (hCG) level SDC: suction dilation and curettage; MTX: methotrexate; EMA-CO: etoposide, methotrexate, actinomycin D, cyclophosphamide, vincristine

Timeline	hCG level (mIU/mL)	Reference range (mIU/mL)
Four weeks post SDC	185	<5
Five weeks post SDC	240	<5
Six weeks post SDC	325	<5
Two weeks post MTX	365	<5
Two weeks post EMA-CO	219	<5

She was referred to oncology due to persistently elevated hCG despite methotrexate, with suspected gestational trophoblastic neoplasm, and underwent four cycles of multi-agent chemotherapy (etoposide, methotrexate, actinomycin D, cyclophosphamide, vincristine (EMA-CO)). There were no features of metastatic disease on the CT chest/abdomen/pelvis. Even though a transient suboptimal reduction of hCG was detected, it remained significantly elevated two weeks after the fourth cycle (Table 1).

**Figure 1:**
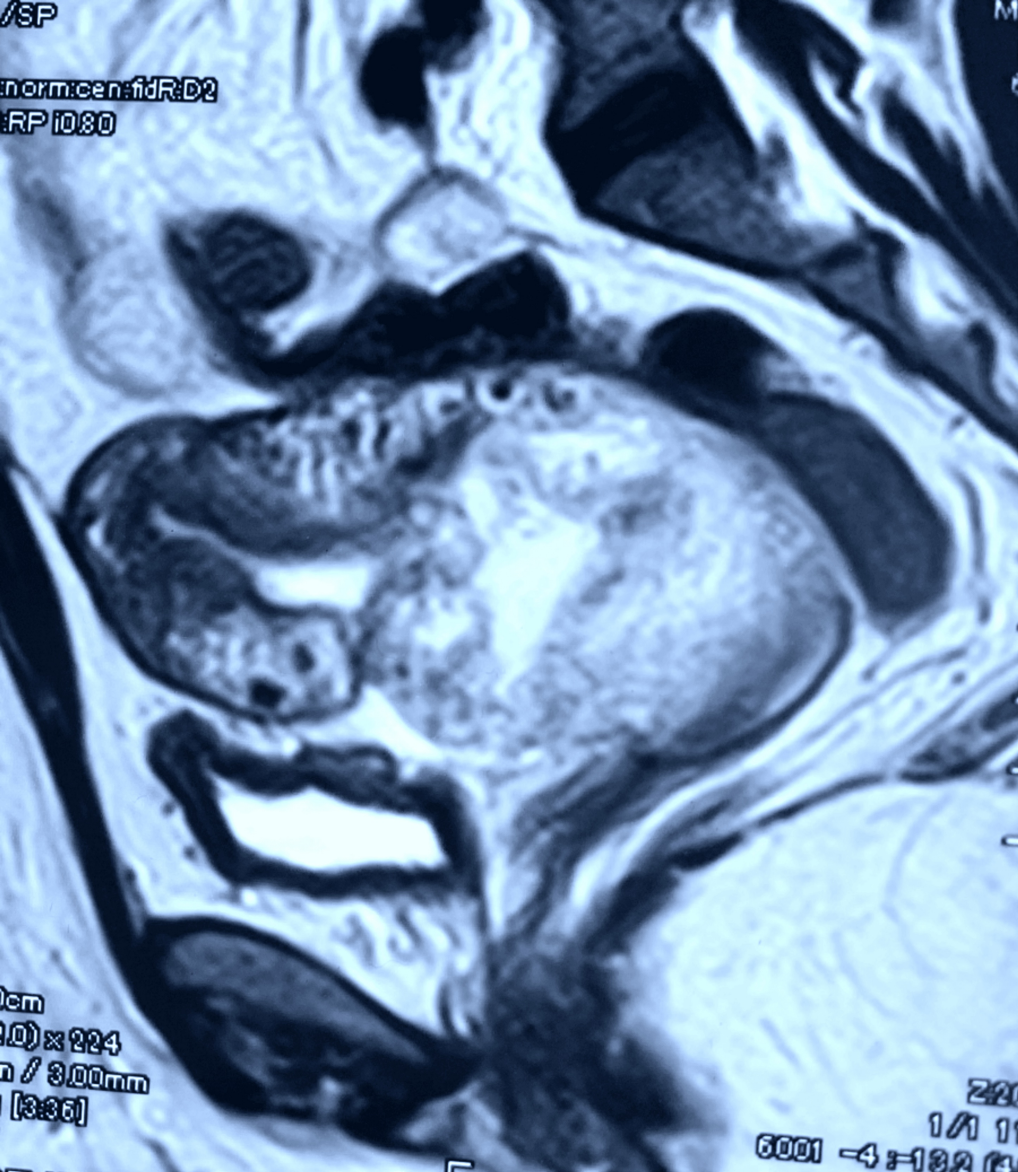
MRI of the pelvis demonstrating infiltration of the lower uterine segment and cervix

**Figure 2:**
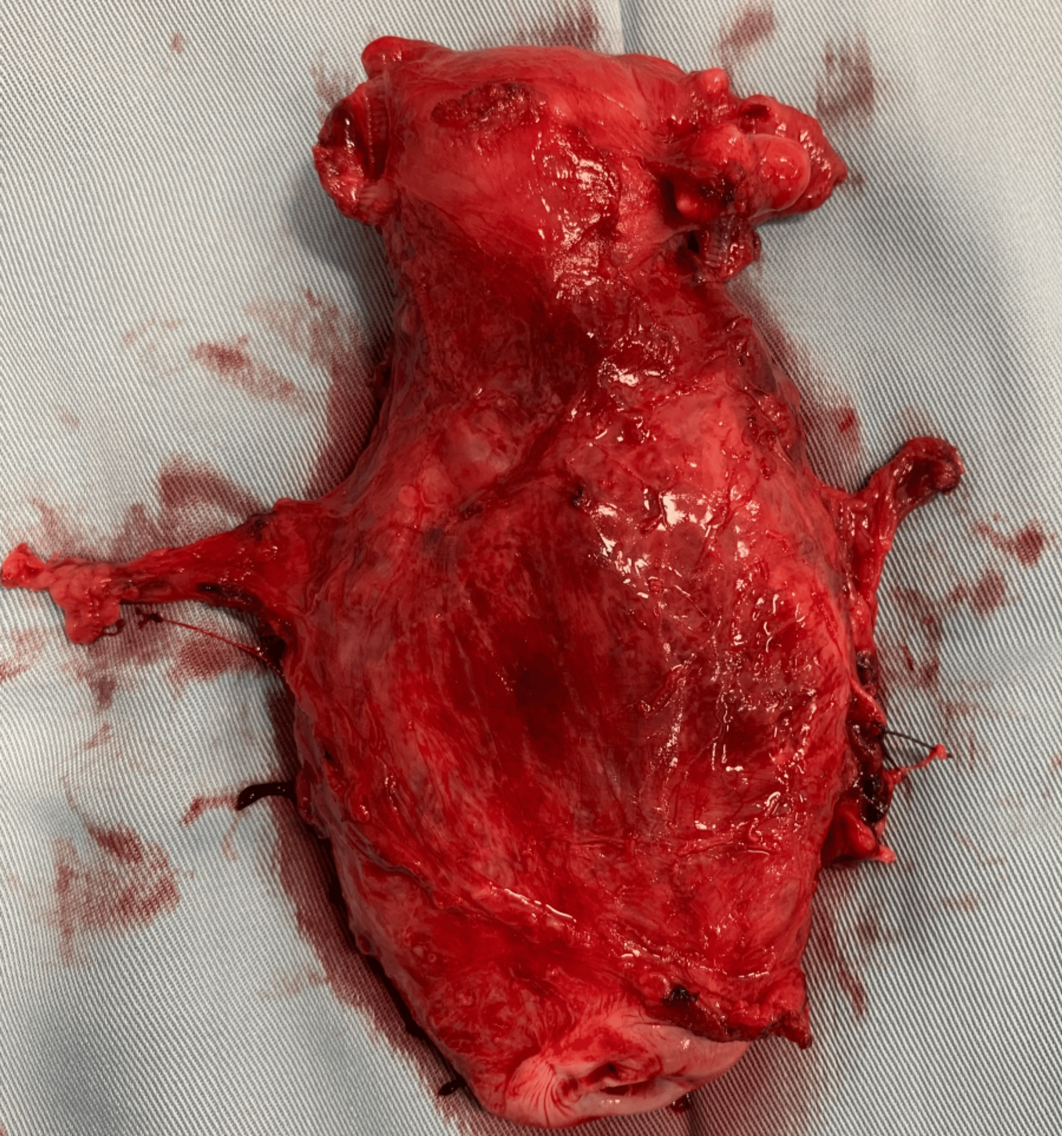
Hysterectomy specimen with the enlarged lower uterine segment

The subsequent MRI of the pelvis revealed infiltration of the lower segment of the uterus and cervix without pelvic metastasis or organ invasion (Figure 1). This radiological finding led to the decision to perform a modified radical hysterectomy with ovarian preservation (Figure 2).

Histological examination revealed a distended lower uterine segment and cervix with predominant mononuclear cells and multinucleated giant cells without evidence of necrosis or mitosis, favoring a diagnosis of exaggerated placental site reaction (EPS). The parametrium, all resection margins, and lymph nodes were free of disease.

The patient had an uneventful recovery, and hCG was undetectable four weeks following the surgery.

## Discussion

GTDs encompass a diverse range of heterogeneous pathologies with varying clinical courses and outcomes. While more common molar pregnancies and choriocarcinoma present with an acute clinical presentation, rarer PSTT, ETT, placental site nodule (PSN), and EPS follow a more indolent clinical course [4,5].

Intermediate trophoblasts play a vital role in placentation by infiltrating the decidua, myometrium, and spiral vessels, thereby creating a low-resistance fetomaternal circulation. Persistence of intermediate trophoblasts can give rise to PSTT, PSN, and EPS [6]. Often, endometrial samples are not adequate to differentiate between EPS and other entities, and they are often diagnosed in hysterectomy specimens [7]. Mitosis and necrosis are often absent in EPS, while this is not the case in PSTT. Although PSN is considered benign, 10%-15% of the cases can coexist or develop into PSTT or ETT [8].

Due to chemoresistance, PSTT, ETT, and PSN are usually managed by surgical excision. Therefore, hysterectomy plays a central part in their management [8,9]. However, conservative management with clinical follow-up and monitoring of hCG could be conducted in EPS, as they can be self-resolving [7]. However, this could be practically difficult as most of the EPS are diagnosed from postoperative hysterectomy specimens [10].

In our case, the combination of a preceding miscarriage, low persistent elevation of hCG, and an extensively infiltrating uterine lesion on MRI was more in favor of a neoplastic disease such as PSTT or placental site nodule, whereas suboptimal reduction of hCG was less in favor of choriocarcinoma. In the background of a grave-looking invasive disease with persistently elevated hCG, the possibility of EPS could be easily overlooked [10]. Considering the challenges in differentiating PSTT, a disease with metastatic potential, from EPS, surgical excision should be considered in the setting of rising or plateau hCG, and there is radiological evidence of possible invasive disease.

## Conclusions

EPS is a benign reactive condition that should be considered an important differential diagnosis in patients presenting with uterine lesions and persistently elevated hCG levels. However, diagnosis of EPS could prove to be a challenge due to its rarity and the close mimicry of clinical and radiological features of gestational trophoblastic neoplasms. Unfamiliarity of clinicians and pathologists with this condition could also be a cause for lower rates of diagnosis. Correct diagnosis of EPS before treatment is crucial, as this disease could potentially be managed conservatively. A preoperative biopsy could be an important step towards differentiating EPS from other gestational trophoblastic neoplasms, even though accuracy could be subject to the size and site of the sample in a large-volume lesion. Therefore, in instances where diagnosis is unclear and/or the possibility of a malignant pathology is suspected, surgical excision might be a safer option.
